# How Well Is Blood Phenylalanine Controlled in Maternal PKU in Europe? Results from 102 Pregnancies

**DOI:** 10.3390/nu18132136

**Published:** 2026-07-02

**Authors:** Alex Pinto, Kirsten Ahring, Manuela Ferreira Almeida, Catherine Ashmore, Sarah Bailey, Amaya Bélanger-Quintana, Alberto Burlina, Duncan Cole, Clare Dale, Anne Daly, Esther van Dam, Charlotte Dawson, Sharon Evans, Sarah Firman, Suzanne Ford, Diane Green, Tarekegn Geberhiwot, Yteke Hoekstra, Sarah Howe, Fatma Ilgaz, Christian Loro, Nicola McStravick, Radha Ramachandran, Katie Rawlins, Louise Robertson, Júlio César Rocha, Iris Rodenburg, Danja Schulenburg-Brand, Francjan J. van Spronsen, Gisela Wilcox, Alison Woodall, Anita MacDonald

**Affiliations:** 1 Birmingham Children’s Hospital, Steelhouse Lane, Birmingham B4 6NH, UK; catherine.ashmore@nhs.net (C.A.); a.daly3@nhs.net (A.D.); sharon.morris6@nhs.net (S.E.); anita.macdonald@nhs.net (A.M.); 2 Departments of Paediatrics and Clinical Genetics, PKU Clinic, Copenhagen University Hospital, Rigshospitalet, Blegdamsvej 9, 2100 Copenhagen, Denmark; kirsten.ahring@regionh.dk; 3 Centro de Genética Médica, Unidade Local de Saúde de Santo António, E.P.E. (ULSSA), 4099-028 Porto, Portugal; manuela.almeida@ulssa.min-saude.pt; 4 Reference Centre of Inherited Metabolic Diseases, Unidade Local de Saúde de Santo António, E.P.E. (ULSSA), 4099-001 Porto, Portugal; 5 Unit for Multidisciplinary Research in Biomedicine, ITR—Laboratory for Integrative and Translational Research in Population Health, Abel Salazar Institute of Biomedical Sciences, ICBAS—School of Medicine and Biomedical Sciences, University of Porto-UMIB/ICBAS/UP, 4050-313 Porto, Portugal; 6 All Wales Inherited Metabolic Disease Service, University Hospital of Wales, Heath Park Way, Cardiff CF14 4XW, UK; sarah.bailey3@wales.nhs.uk (S.B.); duncan.cole@wales.nhs.uk (D.C.); danja.schulenburg-brand@wales.nhs.uk (D.S.-B.); 7 Unidad de Enfermedades Metabólicas Congénitas, Hospital Universitario Ramón y Cajal, 28034 Madrid, Spain; amaya.belanger@salud.madrid.org; 8 Division of Inherited Metabolic Diseases, Reference Centre Expanded Newborn Screening, Department of Women’s and Children’s Health, University Hospital, 35128 Padova, Italy; alberto.burlina@unipd.it; 9 University Hospital Birmingham, National Health Service (NHS) Foundation Trust, Mindelsohn Way, Birmingham B15 2GW, UK; clare.dale@uhb.nhs.uk (C.D.); charlotte.dawson@uhb.nhs.uk (C.D.); tarekegn.geberhiwot@uhb.nhs.uk (T.G.); sarah.howe@uhb.nhs.uk (S.H.); louise.robertson@uhb.nhs.uk (L.R.); 10 Division of Metabolic Diseases, Beatrix Children’s Hospital, University Medical Centre Groningen, University of Groningen, Hanzeplein 1, 9700 RB Groningen, The Netherlands; e.van.dam@umcg.nl (E.v.D.); y.hoekstra@umcg.nl (Y.H.); i.l.rodenburg@umcg.nl (I.R.); f.j.van.spronsen@umcg.nl (F.J.v.S.); 11 Guy’s & St Thomas’ NHS Foundation Trust, Westminster Bridge Rd, London SE1 7EH, UK; sarah.firman1@nhs.net (S.F.); radharamachandran@nhs.net (R.R.); katie.rawlins@gstt.nhs.uk (K.R.); 12 North Bristol Trust, Southmead Rd, Bristol BS10 5NB, UK; suzanne.ford@nbt.nhs.uk; 13 Salford Royal NHS Foundation Trust, Stott Ln, Salford M6 8HD, UK; diane.green@srft.nhs.uk (D.G.); gisela.wilcox@manchester.ac.uk (G.W.); alison.woodall@nca.nhs.uk (A.W.); 14 Department of Nutrition and Dietetics, Faculty of Health Sciences, Hacettepe University, 06100 Ankara, Turkey; fatmacelik86@gmail.com; 15 Division of Dietetic and Clinical Nutrition, Department of Medicine (DIMED), University Hospital of Padua, 35128 Padova, Italy; christian.loro@aopd.veneto.it; 16 The Royal Hospitals Belfast, Belfast Health and Social Care Trust, Grosvenor Road, Belfast BT12 6BA, UK; nicola.mcstravick@belfasttrust.hscni.net; 17 Nutrition and Metabolism, NOVA Medical School|Faculdade de Ciências Médicas NMS|FCM, Universidade Nova de Lisboa, 1169-056 Lisboa, Portugal; rochajc@nms.unl.pt; 18 Centro de Investigação em Tecnologias e Serviços de Saúde (CINTESIS), NOVA Medical School|Faculdade de Ciências Médicas NMS|FCM, Universidade Nova de Lisboa, 1169-056 Lisboa, Portugal; 19 Reference Centre of Inherited Metabolic Diseases, Unidade Local de Saúde São José, 1169-045 Lisboa, Portugal; 20 Comprehensive Health Research Centre (CHRC), NOVA Medical School|Faculdade de Ciências Médicas NMS|FCM, Universidade Nova de Lisboa, 1169-056 Lisboa, Portugal

**Keywords:** phenylketonuria, PKU, natural protein intake, protein equivalent from protein substitute intake, total protein intake, phenylalanine intake

## Abstract

**Background/Objectives**: In phenylketonuria (PKU), high blood phenylalanine (Phe) levels during pregnancy negatively influence foetal organogenesis and growth, leading to maternal PKU syndrome. Pregnancies must be carefully planned in order to maintain blood Phe levels ≤ 360 µmol/L pre-conception and throughout pregnancy. Our aim was to study metabolic control in PKU pregnancies across Europe. **Methods**: Eleven centres managing PKU participated. Data on blood Phe levels (µmol/L), natural protein intake (g/day), protein substitute intake (g/day) and maternal weight (kg) during pregnancy were collected retrospectively from dietetic records between 2012 and 2018. **Results**: In total, 84 female patients with PKU, accounting for 102 pregnancies (mean age: 30.4 ± 4.8 years), participated. Of these, 7 had hyperphenylalaninemia (HPA), 26 had mild PKU, 55 had classical PKU and 14 were unclassified. Sapropterin was prescribed in two pregnancies. Only 27% (28/102) of pregnancies successfully achieved consistent blood Phe levels ≤ 360 µmol/L for at least 2 weeks pre-conception. During pregnancy, 88% of blood Phe levels were ≤360 µmol/L, with a mean Phe of 229 ± 65 µmol/L. The mean number of blood Phe samples was 60 (1.5 per week) per pregnancy. In pre-pregnancy, over a mean of 2.9 years, only 35% of blood Phe levels were ≤360 µmol/L and 61% were <600 µmol/L. Post-pregnancy, over a mean of 2.8 years, 43% of Phe levels were <600 µmol/L with mean Phe 462 ± 226 µmol/L and 724 ± 230 µmol/L, respectively. 25% (25/102) had no levels performed post-pregnancy (mean of 2.8 ± 1.6 years) compared to 7% (7/102) pre-pregnancy (mean of 2.9 ± 1.5 years). Mean prescribed Phe intake pre-/during/post-pregnancy was 810 ± 721 vs. 787 ± 552 vs. 1110 ± 722 mg/day. Natural protein intake was 17 ± 15 vs. 17 ± 11 vs. 23 ± 15 g/day. Protein equivalent from protein substitute intake was 57 ± 21 vs. 66 ± 16 vs. 50 ± 23 g/day and total protein remained stable, 73 ± 14 vs. 83 ± 14 vs. 71 ± 19 g/day (1.1 ± 0.3 vs. 1.1 ± 0.4 vs. 1.0 ± 0.4 g/kg/day). **Conclusions**: Although a high level of metabolic control was maintained during pregnancy, fewer than 30% of pregnancies achieved constant Phe levels ≤ 360 µmol/L prior to conception, with minimal monitoring post-pregnancy. The long-term impact on the offspring remains unknown and requires further investigation.

## 1. Introduction

Maternal phenylketonuria syndrome (MPKUS) is a preventable teratogenic disorder resulting from foetal exposure to elevated maternal phenylalanine (Phe) concentrations [1]. Excess Phe disrupts organogenesis and foetal growth, with the developing brain particularly vulnerable. First recognised by Dent in 1956 [2,3] and formally defined by Lenke and Levy in 1980 [4], MPKUS is characterised by a consistent spectrum of abnormalities, including low birth weight, microcephaly, congenital heart defects, facial dysmorphism, neurocognitive impairment, prematurity, postnatal growth restriction, and neurological deficits [5]. Proposed mechanisms include oxidative stress, metabolic disequilibrium, and disruption of key cellular signalling pathways [6].

Rigorous metabolic control is therefore essential for women with phenylketonuria (PKU) of reproductive age [7]. When maternal Phe is maintained within 120–360 µmol/L before conception or by ~8 weeks’ gestation, foetal outcomes approximate those of the general population, with a 3–5% congenital anomaly risk [5,6,8,9]. In unplanned pregnancies, immediate and sustained Phe reduction is required, with rapid stabilisation, ideally within seven days, which is critical to limiting early teratogenic exposure [10].

Dietary management tailored to individual Phe tolerance remains central to preventing MPKUS. In classical PKU, natural protein tolerance is typically <5 g/day (≈250 mg/day Phe) in the first trimester but increases as pregnancy progresses due to rising protein requirements and foetal phenylalanine hydroxylase (PAH) activity. Protein deposition peaks in the third trimester, with corresponding increases in natural and total protein intake, although tolerance varies widely by PKU severity, maternal weight, foetal genotype, and overall energy intake [11,12,13]. Low-Phe protein substitutes are required throughout pregnancy, and an intake of ≥6 g/day tyrosine is generally achieved when the full prescribed dose is consumed [10]. The revised 2025 European PKU guidelines recommend maintaining blood Phe between 120 and 360 µmol/L for at least two weeks pre-conception and by 8–10 weeks’ gestation [10]. Some centres adopt lower upper thresholds (<300 or <240 µmol/L), although no evidence supports improved outcomes [7]. Conversely, blood Phe < 120 µmol/L may increase the risk of intrauterine growth restriction [14], consistent with broader foetal programming data [15].

Because optimal outcomes depend on achieving metabolic control before conception, proactive reproductive planning and systematic contraception counselling are essential for all women with PKU [16]. Long-term observational data consistently demonstrate the importance of pre-conception control [5,10]. Women adhering to a pre-conception diet have significantly lower mean Phe during pregnancy (203.5 ± 58 vs. 269 ± 115 µmol/L; *p* = 0.0003) [17]. Yet implementation remains inconsistent: only half of pregnancies in historical UK registry data were managed with a pre-conception diet [18], and just 26% (148/574) of women in an international cohort began pregnancy under dietary control [5,18].

Diet discontinuation during reproductive years is common due to limited awareness of MPKUS risks and the difficulty of sustaining strict adherence [19]. Reinstating dietary treatment in adulthood is challenging, and the transition from paediatric to adult services is a recognised point of vulnerability [20,21]. Pregnancy introduces additional barriers, nausea, vomiting, food aversions, and the high cost of special low-protein foods, which further compromise control [22,23]. Our European multicentre study of 1322 individuals showed that Phe concentrations increase with age and that only a minority of women of reproductive age achieve recommended targets [24], placing many at risk if pregnancy occurs without prior optimisation.

This study, therefore, examines metabolic control, dietary intake, and blood Phe monitoring in women with PKU before, during, and after pregnancy. While metabolic control during pregnancy has been repeatedly described, we assess whether outcomes have improved with newer dietetic resources and contemporary practice. Importantly, we also analyse metabolic control and monitoring in the post-pregnancy period, a phase for which published data are scarce. This novel aspect provides new insight into post-partum management within a large European multicentre cohort.

## 2. Materials and Methods

### 2.1. Participating Centres

Eleven European centres providing follow-up care for patients with PKU contributed data to this study. Dietitians and clinicians from the European Nutritionist Expert Panel on PKU (ENEP) and the British Inherited Metabolic Diseases Dietitians Group (BIMDG-DG) participated in data collection. Contributing centres were: A (Copenhagen, Denmark), B (Madrid, Spain), C (Groningen, The Netherlands), D (Padova, Italy), E (Porto, Portugal), F (Belfast, UK), G (Bristol, UK), H (Birmingham, UK), I (Cardiff, UK), J (London, UK), and K (Manchester, UK). All UK centres provided adult-only services, whereas the non-UK European centres delivered care across both paediatric and adult populations.

### 2.2. Patient Selection

Inclusion criteria were women with PKU, identified through newborn screening, diagnosed and initiating treatment by the age of 3 months, management with a Phe-restricted diet and/or adjunct pharmacological therapies, and who experienced at least one full pregnancy between 2012 and 2018.

Exclusion criteria were late diagnosis (age > 3 months of age), delayed initiation of treatment (age > 3 months of age), the presence of a co-existing condition likely to adversely affect metabolic control (e.g., leukaemia), males, and women who did not have a pregnancy during the study period.

### 2.3. Study Design and Data Collection

This multicentre, longitudinal retrospective study collected data on metabolic control and dietary intake in women with PKU before, during, and after pregnancy between 2012 and 2018. Maternal age, PAH variants (when available), and diagnostic Phe concentrations, used to classify PKU severity when genotype data were unavailable, were recorded. Biochemical variables included blood Phe and tyrosine concentrations, as well as the timing of each blood sample.

Dietary intake was assessed using prescribed and recorded intakes of natural protein, total protein, and protein equivalent from protein substitutes. The type of protein substitute used (L-amino acids [L-AA], glycomacropeptide [GMP], or large neutral amino acids) was documented. Anthropometric data (weight and height) and details of pharmacological treatment, including dose during pregnancy, were also extracted. Centres additionally reported the dietary system used to allocate Phe (exchange system versus calculation of total dietary Phe), with most using an exchange-based approach and others calculating total dietary Phe directly.

PKU severity was classified using the BIOPKU database [25] based on available PAH variants; when variant data were unavailable, diagnostic Phe concentrations were used. All data were extracted by A.P. from dietetic and medical records at each participating centre.

### 2.4. Statistical Analysis

There was no sample size calculation, as all eligible patients were invited to participate. The primary outcomes were blood Phe concentrations and dietary intake, including natural protein and protein equivalent from protein substitutes.

Quantitative data and continuous variables were summarised using mean (±standard deviation [SD]) or median (range), while categorical variables were reported as absolute frequencies or percentages. The one-way ANOVA method was used to analyse the difference between pre-, during and post-pregnancy with different PKU severities. All statistical analyses were conducted using GraphPad PRISM (Version 10.1.0, 18 October 2023; GraphPad Software, Boston, MA, USA).

### 2.5. Ethical Aspects

In non-UK centres, ethical approval was obtained in each centre individually. In the UK, ethical approval was granted by the West Midlands Black Country Research Ethics Committee (reference 18/WM/0188) and the Integrated Research Application System (IRAS; number 237853, approved 18 June 2018). The ‘Declaration of Helsinki’ (64th World Medical Association General Assembly, Fortaleza, Brazil, October 2013) and Good Clinical Practice guidelines were followed during the study.

## 3. Results

### 3.1. Treatment Centres and Subject Characteristics

Data were obtained for 84 women with PKU across 102 pregnancies. Data collection was completed in 11 centres, including six in the UK and five in mainland Europe (Table 1; Supplementary Table S1).

The mean maternal age at pregnancy was 30.4 ± 4.8 years. Based on available genotype or diagnostic Phe concentrations, seven pregnancies were from women with hyperphenylalaninaemia (HPA), 26 were mild PKU, and 55 were classical PKU. Fourteen women lacked PAH variant data or diagnostic Phe concentrations and could not be classified.

Table 2 presents data on the type of treatment used during pregnancy in different centres.

Sapropterin was prescribed in two pregnancies only (doses: 15 mg/kg and 11 mg/kg). Dietary intake data were available for all pregnancies. L-AA and/or GMP protein substitutes were used: *n* = 2 pregnancies were managed with GMP alone, *n* = 5 with a combination of GMP and L-AA, and *n* = 93 with L-AA only. No pregnancy was managed with large neutral amino acids. Two pregnancies from women with HPA in a single treatment centre did not require dietary restriction.

All centres, except Centres A and D, permitted fruit and vegetables containing ≤75 mg Phe per 100 g (excluding potatoes) without calculation or measurement.

### 3.2. Overall Data on Metabolic Control

Pre-pregnancy median blood Phe concentration was 574 µmol/L (range 6–1880), with a mean of 462 ± 226 µmol/L over a mean pre-pregnancy interval of 2.9 ± 1.5 years. Blood tyrosine concentrations (*n* = 2260 blood spots) had a mean of 61 ± 31 µmol/L and a median of 54 µmol/L (range 4–420) (Table 3).

During pregnancy, mean blood Phe levels were 229 ± 65 µmol/L and a median of 190 µmol/L (range 6–1590).

Post-pregnancy blood Phe concentrations were higher. The median post-pregnancy Phe concentration was 576 µmol/L (range 20–2208), with a mean of 724 ± 230 µmol/L. Post-pregnancy blood tyrosine concentrations (*n* = 1264 blood spots) had a mean of 61 ± 32 µmol/L and a median of 55 µmol/L (range 11–627).

**Figure 1:**
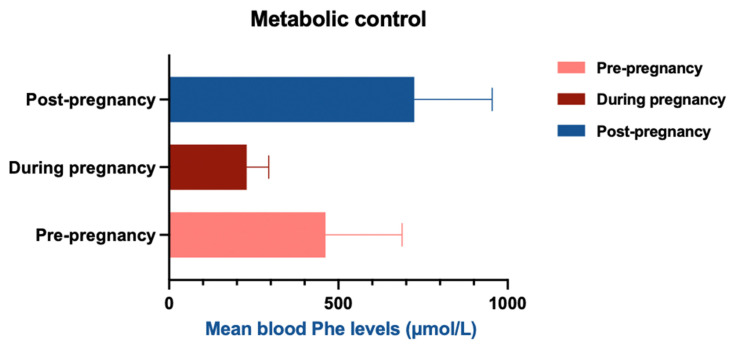
Mean blood Phe levels during the study period.

Figure 1 presents mean blood Phe levels pre-, during and post-pregnancy.

Only 27% (*n* = 28/102) of pregnancies achieved consistently stable pre-conception blood Phe concentrations within the recommended target range (120–360 µmol/L) for at least two weeks before conception (non-UK centres: 18% vs. 33% in UK centres). When the analysis was broadened to include individuals aiming for lower Phe concentrations over a longer pre-conception interval, during a mean of 2.9 years, 35% of pre-pregnancy blood Phe measurements were <360 µmol/L.

During pregnancy, 88% of blood Phe measurements were <360 µmol/L. Mean blood tyrosine (recommended reference range: 40–120 µmol/L) concentration, based on 5477 blood spot samples, was 55 ± 30 µmol/L (median 48 µmol/L; range 9–314). Tyrosine supplementation was prescribed in 10 UK pregnancies.

A mean of 60 blood Phe samples (range 5–135) were collected per pregnancy, corresponding to approximately 1.5 blood spot measurements per week.

Twenty-five per cent of pregnancies did not have any blood Phe levels measured post-pregnancy (mean 2.8 ± 1.6y), compared to only 7% of patients pre-pregnancy (mean 2.9 ± 1.5y).

### 3.3. Metabolic Control by PKU Severity

Table 4 presents blood Phe levels pre, post and during pregnancy by PKU severity.

During pregnancy the results are similar for all PKU severities. However, pre and post pregnancy there are notable changes with a lower percentage of Phe levels within target range for classical PKU and a higher variation in blood Phe demonstrated by increased standard deviation and maximum blood Phe levels. 

### 3.4. Dietary Intake

Dietary intake data were available for all pregnancies and are summarised in Table 5. Although dietary information was complete for all women during pregnancy, data availability decreased to 65 pregnancies in the pre-conception period and to 53 post-pregnancy.

Mean prescribed Phe and natural protein intakes remained largely unchanged from pre- to during pregnancy (Phe: 810 vs. 787 mg/day; mean difference: 23 mg/day; ~3%; natural protein: 17 vs. 17 g/day; no change). In contrast, protein equivalent from protein substitute intake increased during pregnancy compared to pre-conception (57 vs. 66 g/day; mean difference: 9 g/day; ~16%), resulting in a ~14% increase in total protein intake (73 vs. 83 g/day; mean difference: +10 g/day). However, total protein intake per kg body weight remained stable at a mean of 1.1 g/kg/day.

### 3.5. Anthropometry

Pre-pregnancy mean weight was 70 ± 17 kg, during 72 ± 16 kg and 73 ± 18 post-pregnancy. Mean height was 164 ± 6, 163 ± 12 and 164 ± 6 cm pre, during and post-pregnancy. Mean BMI was 26 ± 6, 28 ± 16 and 27 ± 6 pre, during and post-pregnancy (Supplementary Table S2).

The mean increase in weight from the pre-pregnancy period to during pregnancy was 3 ± 8 kg, with a median change of 3 kg (range –13 to 20). Baseline and end-of-pregnancy measurements were generally unavailable, so the data are based on annual assessments. This limitation substantially restricts the interpretability of the weight-change findings.

## 4. Discussion

This retrospective multicentre study of 102 pregnancies across 11 European centres demonstrates persistent gaps in pre- and post-pregnancy metabolic management in women with PKU. Despite longstanding guideline recommendations to maintain maternal Phe concentrations within 120–360 µmol/L for at least two weeks before conception, only 27% achieved this target, indicating that many pregnancies were unplanned and aligning with previous reports [19]. Once pregnancy commenced, metabolic control improved substantially, with 88% of Phe measurements within range under intensive monitoring. However, monitoring and dietary data were markedly reduced before and after pregnancy, with 25% of women having no post-pregnancy Phe measurements. Although PKU severity did not influence metabolic control during pregnancy, lower pre- and post-pregnancy target attainment was associated with greater severity, consistent with broader multicentre findings [24].

Metabolic control during pregnancy was comparable to earlier cohorts [5,16,17], despite advances in dietary products and special low-protein foods [26,27,28]. Women who were managed with a diet-only treatment continue to face greater challenges than those responsive to sapropterin or pegvaliase [29,30,31,32,33]. Most women ultimately achieved good metabolic control during pregnancy, including those with longstanding adherence difficulties, reflecting the strong motivational effect of foetal risk. Nevertheless, achieving target Phe concentrations before conception or by 8–10 weeks’ gestation remains essential to prevent MPKUS [10,34].

In this cohort, prescribed natural protein intake appeared broadly similar before and during pregnancy; however, interpretation requires caution, as adherence and metabolic control are typically higher during pregnancy and may therefore yield more reliable data than pre- or post-pregnancy periods characterised by greater non-adherence. Physiologically, natural protein tolerance is expected to increase in the second and third trimesters as protein requirements rise and foetal PAH activity contributes to Phe-to-tyrosine conversion [10]. This increase may be attenuated or absent when the foetus is also affected by PKU and lacks PAH activity, and tolerance can vary between pregnancies in the same woman, reflecting differences in maternal weight, metabolic status, and other physiological factors [19]. Post-pregnancy dietary data were limited, and breastfeeding practices were not collected. Lactation requires an additional 20 g/day of protein during the first six months [10], yet total protein intake decreased after pregnancy in this cohort. Although data are sparse, such a short-fall may place both mother and infant at risk of inadequate nutritional status, potentially compromising breastfeeding and adding psychological strain during an already vulnerable period.

Protein intake from protein substitutes increased modestly during pregnancy in this cohort, but did not quite meet the PKU European guideline recommendations to increase protein substitutes by ~20% in early pregnancy and by a further 30 g/day in the third trimester [10]. Evidence from the Maternal PKU Collaborative Study indicates that higher total protein intake is associated with lower maternal Phe and improved birth outcomes [35,36,37]. However, interpretation of dietary data in this study is constrained by reliance on prescribed rather than actual intake and by limited pre- and post-pregnancy data. Although GMP-based protein substitutes may offer improved tolerability during pregnancy and potential prebiotic benefits, their use was limited in our cohort and evidence in pregnancy remains preliminary [10]. Further research is needed to define their safety, tolerability, and metabolic impact across gestation.

Monitoring practices in maternal PKU also require modernisation. Dried blood spot methodology is slow; it is subject to postal delays, and gives blood Phe concentrations 15–20% lower than venous samples [38,39,40,41,42,43]. New point-of-care technologies offer rapid results and strong correlation with dried blood spots [44], but their integration into pregnancy care requires direct data transmission to clinical teams to ensure safe dietary adjustment.

Only 27% of women achieved optimal metabolic control before conception, a finding with considerable clinical implications given associations between suboptimal pre-conception care, miscarriage (~7%), and lower early-childhood cognitive scores [45]. Structured pre-conception preparation markedly improves metabolic control and offspring outcomes [16,45], and yet unplanned pregnancy remained common (35–54% across regions). Psychosocial, socioeconomic, and cognitive factors, including unstable housing, limited support networks, and reduced intellectual ability, likely contribute to poor preparedness [46,47,48]. Strengthening reproductive planning, contraception counselling, and coordinated care across metabolic, obstetric, and primary-care services is therefore essential.

Women who were not following an optimal low-Phe diet prior to pregnancy were also likely at risk of long-term nutritional deficiencies [49,50]. Prenatal nutrition is a major determinant of maternal and foetal outcomes [51,52], and the rising prevalence of obesity among women of reproductive age adds further metabolic vulnerability through hormonal dysregulation, insulin resistance, and chronic inflammation [53]. Adequate gestational weight gain is essential to support foetal development and prevent maternal catabolism. Although women in this cohort entered pregnancy overweight, their mean gestational weight gain was only 3 kg, substantially below the 7–11.5 kg recommended for this BMI category [54]. Interpretation is limited by sparse, non-standardised data and the absence of baseline and birth-weight measurements. Given that metabolic control was maintained, catabolism is unlikely; however, the restricted dataset prevents firm conclusions.

Post-pregnancy monitoring declined sharply. Although 88% of pregnancy measurements were within target range, this fell to 43% afterwards, and 25% of women performed no monitoring for two years. Although elevated maternal Phe does not affect the breastfed infant, its potential impact on maternal well-being warrants attention. Adults with PKU have higher rates of anxiety and depression [55,56,57], and the postpartum period is a recognised time of vulnerability [58,59]. Targeted postpartum support, including group-based interventions, peer networks, and structured follow-up, may help sustain adherence and prevent loss to follow-up. The absence of offspring neurodevelopmental data remains a major evidence gap.

## 5. Limitations

This study has several important limitations. Early treatment was defined as initiation of a low-protein diet by 3 months of age; however, treatment practices varied across centres and evolved over time. All data were collected retrospectively by multiple clinical teams with differing management approaches, which may have introduced inconsistency. Source data were extracted from a combination of electronic and paper medical and dietetic records, and variability in documentation systems and record quality may have affected data completeness and accuracy.

Blood Phe concentrations were analysed by different laboratories using methods with varying sensitivity, which may have influenced the comparability of metabolic control data. In addition, Phe concentrations measured from dried blood spots are known to be approximately 7–13% lower than venous samples [39,40,41,42,43], suggesting that true blood Phe levels may have been higher than those reported. Target blood Phe ranges also differed between countries during the study period, particularly for older patients, and were not always aligned with European PKU guidelines during non-pregnancy periods [24,60]. These differences may have affected the extent to which women achieved pre-conception dietary targets.

Incomplete genotype and diagnostic Phe data in several centres limited accurate classification of PKU severity and may have introduced bias. The absence of detailed dietary intake data during adulthood, particularly during the pre- and post-conception periods, further restricts interpretation of dietary adherence and its relationship to metabolic control. Available dietary intake data reflected prescribed rather than actual intake, which may not accurately represent real-world behaviour. Moreover, no trimester-specific dietary intake data were available, nor were weight trajectories or trimester-specific metabolic control data, limiting the precision with which dietary patterns and physiological changes could be interpreted. 

Monitoring of both diet and blood Phe outside pregnancy was limited, contrasting with the intensive surveillance typical during pregnancy. This discrepancy complicates direct comparisons and may obscure the true metabolic profile of women with PKU before and after pregnancy. The absence of data on breastfeeding practices or whether infants were born with PKU further constrains the interpretation of maternal–child health relationships. Energy-intake data were also unavailable. Weight measurements were collected only annually; therefore, trimester-specific weight gain and baseline-to-end-of-pregnancy changes could not be assessed. As a result, anthropometric comparisons during pregnancy are imprecise and do not allow firm conclusions about maternal weight gain in women with PKU. No offspring data were collected, leaving the impact of maternal metabolic control on child outcomes unknown.

## 6. Conclusions

Although metabolic control during pregnancy was generally well maintained, fewer than 30% of women achieved recommended Phe concentrations before conception, and post-pregnancy monitoring declined substantially. Sparse dietary data before and after pregnancy limit the interpretation of metabolic patterns across these stages. The long-term implications for offspring and for maternal well-being remain insufficiently understood. There is a clear need for safe adjunct therapies, improved pre-conception support, and structured postpartum follow-up to optimise care for women of reproductive age with PKU.

## Figures and Tables

**Table 1:** Participating centres and patient characteristics.

Centre	Number of Women with PKU	Number of Pregnancies	Number of Women with More Than One Pregnancy During Study Period	% of Pregnancies Achieving Recommended Pre-Conception Blood Phe Levels (Number of Pregnancies)	PKU Classification of Patients on Pre-Conception Diet (Number of Pregnancies)	Classical PKU	Mild PKU	HPA	PKU Severity Classification Data Not Available
A	25	29	4	14% (*n* = 4)	HPA (*n* = 1) Mild PKU (*n* = 1) Classical PKU (*n* = 2)	13	10	6	0
B	1	1	0	100% (*n* = 1)	Classical PKU (*n* = 1)	1	0	0	0
C	4	5	1	20% (*n* = 1)	Mild PKU (*n* = 1)	2	3	0	0
D	1	1	0	0	-	0	1	0	0
E	3	3	0	33% (*n* = 1)	Mild PKU (*n* = 1)	1	2	0	0
F	13	17	3	24% (*n* = 4)	HPA (*n* = 1) Mild PKU (*n* = 3)	9	7	1	0
G	3	4	1	50% (*n* = 2)	Classical PKU (*n* = 2)	3	0	0	1
H	12	15	3	40% (*n* = 6)	N/A (*n* = 3) Classical PKU (*n* = 3)	9	0	0	6
I	3	3	0	67% (*n* = 2)	Classical PKU (*n* = 2)	2	0	0	1
J	4	6	2	50% (*n* = 3)	N/A (*n* = 2) Classical PKU (*n* = 1)	2	1	0	3
K	15	18	3	22% (*n* = 4)	N/A (*n* = 2) Classical PKU (*n* = 2)	13	2	0	3
Total non-UK centres	34	39	5	18% (*n* = 7)	HPA (*n* = 1) Mild PKU (*n* = 3) Classical PKU (*n* = 3)	17	16	6	0
UK centres	50	63	12	33% (*n* = 21)	N/A (*n* = 7) HPA (*n* = 1) Mild PKU (*n* = 3) Classical PKU (*n* = 10)	38	10	1	14
Total	84	102	17	27% (*n* = 28)	N/A (*n* = 7) HPA (*n* = 2) Mild PKU (*n* = 6) Classical PKU (*n* = 13)	55	26	7	14

Abbreviations: PKU—Phenylketonuria; HPA—Hyperphenylalaninaemia; N/A—not available.

**Table 2:** Data on the type of treatment and protein substitute use during pregnancies by centre.

Centre	Number of Patients	Number of Pregnancies	No Dietary Management	Protein Substitute			Sapropterin
				AA Only	GMP + AA	GMP Only	
A	25	29	2	23	4	0	0
B	1	1	0	1	0	0	0
C	4	5	0	5	0	0	1
D	1	1	0	1	0	0	1
E	3	3	0	2	0	1	0
F	13	17	0	17	0	0	0
G	3	4	0	3	1	0	0
H	12	15	0	14	0	1	0
I	3	3	0	3	0	0	0
J	4	6	0	6	0	0	0
K	15	18	0	18	0	0	0
Total	84	102	2	93	5	2	2

Abbreviations: GMP—Glycomacropeptide-based protein substitute; AA—amino acid-based protein substitute.

**Table 3:** Metabolic control pre-/during/post-pregnancy.

	Blood Phe Levels (µmol/L) Mean ± SD Median (Range)	% of Levels Within Target Range (Range)	Blood Tyr Levels (µmol/L) Mean ± SD Median (Range)
Pre-pregnancy (*n* = 2709)	462 ± 226 574 (6–1880)	61% (120–600 µmol/L)	61 ± 31 54 (4–420)
During pregnancy (*n* = 6107)	229 ± 65 190 (6–1590)	88% (120–360 µmol/L)	55 ± 30 48 (9–314)
Post-pregnancy (*n* = 1463)	724 ± 230 576 (20–2208)	43% (120–600 µmol/L)	61 ± 32 55 (11–627)

Abbreviations: Phe—Phenylalanine; Tyr—Tyrosine.

**Table 4:** Metabolic control pre-/during/post-pregnancy by PKU severity.

PKU Severity (Number of Patients)		Pre-Pregnancy	During Pregnancy	Post-Pregnancy	ANOVA
HPA (*n* = 7)	Blood Phe levels (µmol/L) Mean ± SD Median (range)	358 ± 257 293 (17–1342)	246 ± 102 242 (79–652)	390 ± 221 376 (95–1050)	F (2, 614) = 41.27, *p* < 0.0001
	Mean % of levels within target	82% (<600 µmol/L) 61% (<360 µmol/L)	100% (<600 µmol/L) 87% (<360 µmol/L)	79% (<600 µmol/L) 48% (<360 µmol/L)	
	Number of blood Phe spots	133	375	109	
Mild PKU (*n* = 26)	Blood Phe levels (µmol/L) Mean ± SD Median (range)	444 ± 252 408 (44–1669)	229 ± 117 203 (18–1202)	558 ± 330 507 (106–2208)	F (2, 2378) = 487.3, *p* < 0.0001
	Mean % of levels within target	79% (<600 µmol/L) 41% (<360 µmol/L)	98% (<600 µmol/L) 90% (<360 µmol/L)	62% (<600 µmol/L) 31% (<360 µmol/L)	
	Number of blood Phe spots	673	1307	401	
Classical PKU (*n*= 55)	Blood Phe levels (µmol/L) Mean ± SD Median (range)	490 ± 331 438 (16–1880)	230 ± 157 190 (6–1590)	656 ± 458 590 (23–2183)	F (2, 5993) = 1115, *p* < 0.0001
	Mean % of levels within target	68% (<600 µmol/L) 40% (<360 µmol/L)	96% (<600 µmol/L) 87% (<360 µmol/L)	50% (<600 µmol/L) 36% (<360 µmol/L)	
	Number of blood Phe spots	1510	375	907	

Abbreviations: Phe—Phenylalanine; HPA—hyperphenylalaninemia.

**Table 5:** Dietary data pre-, during and post-pregnancy.

Time of Data Collection (*n* of Pregnancies with Dietary Intake Data)	Dietary Intakes							
	Phe (mg/Day)		Natural Protein (g/Day)		Protein Equivalent from Protein Substitute (g/Day)		Total Protein (g/Day)	
	Mean ± SD	Median [Range]	Mean ± SD	Median [Range]	Mean ± SD	Median [Range]	Mean ± SD	Median [Range]
Pre-pregnancy (n = 65)	810 ± 721	550 [150–3000]	17 ± 15	12 [3–61]	57 ± 21	60 [0–100]	73 ± 14	67 [45–140]
During pregnancy (n = 102)	787 ± 552	650 [150–3500]	17 ± 11	15 [3–70]	66 ± 16	64 [24–125]	83 ± 14	82 [56–149]
Post-pregnancy (n = 53)	1110 ± 722	875 [150–3100]	23 ± 15	18 [3–62]	50 ± 23	59 [0–90]	71 ± 19	72 [22–119]

Abbreviations: Phe—phenylalanine; *n*—number; SD—standard deviation.

## Data Availability

The original contributions presented in this study are included in the article/Supplementary Materials. Further inquiries can be directed to the corresponding author.

## Supplementary Materials

The following supporting information can be downloaded at: https://www.mdpi.com/article/10.3390/nu18132136/s1, Table S1: Description of all pregnancies; Table S2: Anthropometric measurements pre, during and post pregnancy.
