# Supplementary material for: How Well Is Blood Phenylalanine Controlled in Maternal PKU in Europe? Results from 102 Pregnancies
Source: Nutrients. 2026 Jul 2;18(13):2136. doi: 10.3390/nu18132136 (PMC13364312; doi:10.3390/nu18132136)
Supplement: Supplementary file 1 [file nutrients-18-02136-s001.zip › nutrients-4368839-supplementary.pdf]

Supplementary Table S1. Description of all pregnancies.

Patient number	Centre	PKU mutation	PKU diagnostic	Pregnancy year	Age at pregnancy	Type of protein substitute	Use of BH4 During pregnancy	Pre-conception levels achieved
**1**	Copenhagen, Denmark	IVS12+1G>A (c.1315+1G>A)<br>I306V (c.916A>G)	HPA	2014	40	AA	No	No
**2**	Copenhagen, Denmark	I306V (c.916A>G)	HPA	2014/2015	37	AA	No	No
**3**	Copenhagen, Denmark	E280K (c.838G>A)<br>R408W (c.1222C>T)	Classical PKU	2012/2013	34	AA	No	No
**4**	Copenhagen, Denmark	IVS12+1G>A (c.1315+1G>A)<br>R158Q (c.473G>A)	Classical PKU	2013/2014	33	AA	No	No
**5**<br>**1st pregnancy**	Copenhagen, Denmark	R408W (c.1222C>T)<br>R408W (c.1222C>T)	Classical PKU	2012/2013	30	AA	No	No
**5**<br>**2nd pregnancy**	Copenhagen, Denmark	R408W (c.1222C>T)<br>R408W (c.1222C>T)	Classical PKU	2016/2017	34	AA	No	No
**6**	Copenhagen, Denmark	IVS12+1G>A (c.1315+1G>A)<br>R408W (c.1222C>T)	Classical PKU	2015	32	AA	No	No
**7**	Copenhagen, Denmark	IVS12+1G>A (c.1315+1G>A)<br>Y414C (c.1241A>G)	Mild PKU	2012	29	AA	No	No
**8**	Copenhagen, Denmark	IVS12+1G>A (c.1315+1G>A)<br>Y414C (c.1241A>G)	Mild PKU	2013/2014	30	AA	No	No
**9**	Copenhagen, Denmark	IVS12+1G>A (c.1315+G>A)<br>?	Mild PKU	2014/2015	30	AA	No	No
**10**	Copenhagen, Denmark	IVS12+1G>A (c.1315+G>A)	Classical PKU	2015/2016	31	AA	No	No
**11**	Copenhagen, Denmark	IVS10-11G>A (c.1066-11G>A)<br>A104D (c.311C>A)	Mild PKU	2013	28	AA	No	Yes
**12**	Copenhagen, Denmark	IVS12+1G>A (c.1315+G>A)<br>R408W (c.1222C>T)	Classical PKU	2016	31	AA	No	No
**13**<br>**1st pregnancy**	Copenhagen, Denmark	R408W (c.1222C>T)<br>Y414C (c.1241A>G)	Mild PKU	2012	26	AA	No	N/A
**13**<br>**2nd pregnancy**	Copenhagen, Denmark	R408W (c.1222C>T)<br>Y414C (c.1241A>G)	Mild PKU	2015/2016	29	AA + GMP	No	No

**14**<br>**1st pregnancy**	Copenhagen, Denmark	P281L<br>Y414C (c.1241A>G)	Mild PKU	2013/2014	27	AA	No	No
**14**<br>**2nd pregnancy**	Copenhagen, Denmark	P281L<br>Y414C (c.1241A>G)	Mild PKU	2016/2017	30	AA	No	No
**15**	Copenhagen, Denmark	IVS12+1G>A (c.1315+G>A)<br>R408W (c.1222C>T)	Classical PKU	2017	34	AA + GMP	No	No
**16**	Copenhagen, Denmark	A104D (c.311C>A)<br>P281L (c.842C>T)	Classical PKU	2015	27	AA	No	No
**17**	Copenhagen, Denmark	IVS12+1G>A (c.1315+G>A)<br>R408W (c.1222C>T)	Classical PKU	2014/2015	26	AA + GMP	No	No
**18**	Copenhagen, Denmark	R176L<br>R408W (c.1222C>T)	HPA	2015	27	No diet	No	No
**19**	Copenhagen, Denmark	IVS12+1G>A (c.1315+G>A)<br>I174T (c.521T>C)	Classical PKU	2015/2016	26	AA	No	No
**20**<br>**1st pregnancy**	Copenhagen, Denmark	n/a	Mild PKU	2014	24	AA	No	No
**20**<br>**2nd pregnancy**	Copenhagen, Denmark	n/a	Mild PKU	2016	26	AA+GMP	No	No
**21**	Copenhagen, Denmark	IVS12+1G>A (c.1315+G>A)<br>A47V	HPA	2015/2016	24	AA	No	No
**22**	Copenhagen, Denmark	L98S (c.293T>C)<br>L98S (c.293T>C)	HPA	2014/2015	22	AA	No	No
**23**	Copenhagen, Denmark	IVS12+1G>A (c.1315+G>A)<br>?	Classical PKU	2018	22	AA	No	Yes
**24**	Copenhagen, Denmark	IVS7+1G>A<br>R408W (c.1222C>T)	Classical PKU	2016	39	AA	No	Yes
**25**	Copenhagen, Denmark	A300S (c.898G>T) / A403V (c.1208C>T)	HPA	2016/2017	32	No diet	No	Yes
**26**	Madrid, Spain	R111X<br>R261Q	Classical PKU	2016-2017	29	AA	No	Yes
**27**	Groningen, The Netherlands	c.1241A>A (pY414C)<br>c.842+5G>A (IVS7+5(g>a)	Mild PKU	2016	34	AA	15mg/kg	Yes
**28**	Groningen, The Netherlands	c.569T>C (Val190Ala)<br>c.782G>A (Arg261Gln)	Mild PKU	2017	31	AA	No	No

Supplementary Table S1. Description of all pregnancies.

**29**<br>**1st pregnancy**	Groningen, The Netherlands	c.814 G>T (p.G272X)<br>C.510-21_665del 177 (IVS5-21del177)	Classical PKU	2013	25	AA	No	No
**29**<br>**2nd pregnancy**	Groningen, The Netherlands	c.814 G>T (p.G272X)<br>C.510-21_665del 177 (IVS5-21del177)	Classical PKU	2015	27	AA	No	No
**30**	Groningen, The Netherlands	c.1315+1G>A<br>c.311C>A (p.Ala104Asp)	Mild PKU	2016	28	AA	No	No
**31**	Padova, Italy	p.L48S<br>p.R261Q	Mild PKU	2015	24	AA	Yes 11mg/kg	No
**32**	Porto, Portugal	p.I65T<br>p.R261Q	Mild PKU	2017	28	AA	No	Yes
**33**	Porto, Portugal	p.R270K<br>?	Mild PKU	2017/2018	27	GMP	No	No
**34**	Porto, Portugal	p.P281L<br>p.P281L	Classical PKU	2015	31	AA	No	No
**35**	Belfast, UK	F299C<br>L348V	Mild PKU	2012/2013	31	AA	No	No
**36**	Belfast, UK	T380M / 165T	HPA	2014/2015	23	AA	No	Yes
**37**	Belfast, UK	N/A	Classical PKU	2015/2016	34	AA	No	No
**38**	Belfast, UK	S273F/?	Classical PKU	2013	38	AA	No	No
**39**	Belfast, UK		Mild PKU	2015	33	AA	No	No
**40**<br>**1st pregnancy**	Belfast, UK	R408W/R158Q	Classical PKU	2012	35	AA	No	N/A
**40**<br>**2nd pregnancy**	Belfast, UK	R408W/R158Q	Classical PKU	2015	38	AA	No	No
**41**<br>**1st pregnancy**	Belfast, UK	R408W/R408W	Classical PKU	2014	22	AA	No	No
**41**<br>**2nd pregnancy**	Belfast, UK	R408W/R408W	Classical PKU	2016	24	AA	No	No
**42**<br>**1st pregnancy**	Belfast, UK	n/A	Mild PKU	2012	35	AA	No	N/A
**42**	Belfast, UK	n/A	Mild PKU	2014	37	AA	No	No

**2nd pregnancy**								
**42**<br>**3rd pregnancy**	Belfast, UK	n/A	Mild PKU	2016	39	AA	No	Yes
**43**	Belfast, UK	R408W / Y414C	Mild PKU	2014	34	AA	No	Yes
**44**	Belfast, UK	L348V / 165T	Mild PKU	2018	27	AA	No	Yes
**45**	Belfast, UK	F39L / IVS12+1G>A	Classical PKU	2018	22	AA	No	No
**46**	Belfast, UK	165T / 165T	Classical PKU	2015	29	AA	No	No
**47**	Belfast, UK	N/A	Classical PKU	2013	31	AA	No	No
**48**<br>**1st pregnancy**	Bristol, UK	c.722G>T p.(Arg241Leu / c.782G>A p.(Arg261Gln)	Classical PKU	2016	30	AA	No	Yes
**48**<br>**2nd pregnancy**	Bristol, UK	c.722G>T p.(Arg241Leu / c.782G>A p.(Arg261Gln)	Classical PKU	2018	32	AA+GMP	No	No
**49**	Bristol, UK	c.194T>C p.(Ile65Thr) /c.331C>T p.(Arg111*)	Classical PKU	2016/2017	33	AA	No	Yes
**50**	Bristol, UK	c..194T>C p.(Ile65Thr)/?	N/A	2016/2017	23	AA	No	No
**51**	Birmingham, UK	N/A	Classical PKU	2018	27	AA	No	No
**52**	Birmingham, UK	c.473G>A p.(Arg158Gln)	Classical PKU	2013	29	AA	No	No
**53**	Birmingham, UK	N/A	N/A	2012/2013	42	AA	No	No
**54**<br>**1st pregnancy**	Birmingham, UK	N/A	N/A	2015/2016	33	AA	No	No
**54**<br>**2nd pregnancy**	Birmingham, UK	N/A	N/A	2018	36	GMP	No	No
**55**	Birmingham, UK	c.117c>G: p.(phe39leu) C.1222C>T;p.(Arg408Trp)	Classical PKU	2017	34	AA	No	Yes
**56**<br>**1st pregnancy**	Birmingham, UK	N/A	N/A	2014/2015	33	AA	No	Yes
**56**<br>**2nd pregnancy**	Birmingham, UK	N/A	N/A	2016/2017	35	AA	No	Yes

Supplementary Table S1. Description of all pregnancies.

**57**	Birmingham, UK	c.194T>C (p.Ile65Thr)<br>c.1222C>T (p.Arg408Trp)	Classical PKU	2012	34	AA	No	N/A
**58**	Birmingham, UK	c.1222C>T (p.Arg408Trp)<br>?	Classical PKU	2016	27	AA	No	No
**59**	Birmingham, UK	p.(Pro281Leu)<br>c.1315+1G>A (IVS12+1G>A)	Classical PKU	2018	32	AA	No	Yes
**60**	Birmingham, UK	c.782G>A p.(Arg261Gln)<br>Del Ex6	Classical PKU	2018	29	AA	No	Yes
**61**	Birmingham, UK	c.117 C>G p.(Phe39Leu)<br>?	N/A	2016	32	AA	No	Yes
**62<br>1st pregnancy**	Birmingham, UK	c.165del p.(Phe55Leufs*6)<br>Deletion of 5'UTR, exon 1 and exon 2*	Classical PKU	2012/2013	36	AA	No	No
**62<br>2nd pregnancy**	Birmingham, UK	c.165del p.(Phe55Leufs*6)<br>Deletion of 5'UTR, exon 1 and exon 2*	Classical PKU	2014	38	AA	No	No
**63**	Cardiff, UK	N/A	N/A	2014	31	AA	No	No
**64**	Cardiff, UK	N/A	Classical PKU	2014	32	AA	No	Yes
**65**	Cardiff, UK	N/A	Classical PKU	2018	29	AA	No	Yes
**66**	London, UK	c.1222C>T p.(Arg408Trp) /<br>c.1241A>G p.(Tyr414Cys)	Mild PKU	2014	23	AA	No	No
**67**	London, UK	N/A	N/A	2016/2017	31	AA	No	No
**68<br>1st pregnancy**	London, UK	N/A	N/A	2014/2015	28	AA	No	Yes
**68<br>2nd pregnancy**	London, UK	N/A	N/A	2017	31	AA	No	Yes
**69<br>1st pregnancy**	London, UK	c.664_665del p.(Asp222*) /<br>c.1315+1G>A p.?	Classical PKU	2014	34	AA	No	Yes
**69<br>2nd pregnancy**	London, UK	c.664_665del p.(Asp222*) /<br>c.1315+1G>A p.?	Classical PKU	2016/2017	36	AA	No	No
**70**	Manchester, UK	c.1066-11G>A p.? / c.<br>1223G>A p. (Arg408Gin)	Mild PKU	2014	32	AA	No	No

**71**	Manchester, UK	c. 728G>A p. (Arg243Gln) / c. 728G>A p. (Arg243Gln)	Classical PKU	2016	29	AA	No	Yes
**72**	Manchester, UK	c. 838G>A p. (Glu280Lys) / c. 1042C>G p. (Leu348Val)	Classical PKU	2014	35	AA	No	No
**73 1st pregnancy**	Manchester, UK	c.838G>Ap.(Glu280Lys) / c.838G>Ap.(Glu280Lys)	Classical PKU	2014	19	AA	No	No
**73 2nd pregnancy**	Manchester, UK	c.838G>Ap.(Glu280Lys) / c.838G>Ap.(Glu280Lys)	Classical PKU	2017	22	AA	No	No
**74**	Manchester, UK	N/A	N/A	2018	32	AA	No	Yes
**75**	Manchester, UK	c. 664_665del p. (Asp222*) / c. 727C>T p. (Arg243*)	Classical PKU	2014	39	AA	No	No
**76**	Manchester, UK	N/A	N/A	2016	32	AA	No	No
**77**	Manchester, UK	N/A	N/A	2017/2018	29	AA	No	Yes
**78**	Manchester, UK	c.194T>C p+J43:K44.(Ile65Thr) / c.1315+1G>A p.?	Classical PKU	2012/2013	27	AA	No	No
**79**	Manchester, UK	N/A	Classical PKU	2018	22	AA	No	No
**80**	Manchester, UK	c.1222C>T p.(Arg408Trp) / c. 1223G>A p. (Arg408Gin).	Mild PKU	2015	32	AA	No	No
**81**	Manchester, UK	I65T/ IVS7nt+1	Classical PKU	2012	25	AA	No	n/a
**82 1st pregnancy**	Manchester, UK	IVS12nt+1 / I65T	Classical PKU	2012	30	AA	No	n/a
**82 2nd pregnancy**	Manchester, UK	IVS12nt+1 / I65T	Classical PKU	2015	33	AA	No	Yes
**83**	Manchester, UK	c. 782G>Ap. (Arg261Gin) / c. 1315+1G>A p. ?	Classical PKU	2012	36	AA	No	n/A
**84 1st pregnancy**	Manchester, UK	c. 194T>C p. (Ile65Thr) / c. 472C>T p. (Arg158Trp)	Classical PKU	2013	26	AA	No	No
**84 2nd pregnancy**	Manchester, UK	c. 194T>C p. (Ile65Thr) / c. 472C>T p. (Arg158Trp)	Classical PKU	2018	31	AA	No	No

Abbreviations: PKU, Phenylketonuria; HPA, Hyperphenylalaninemia, AA, amino-acid based protein substitue; GMP, glycomacropeptide based protein substitute; BH4, sapropterin; NA, not available.

Supplementary Table S1. Description of all pregnancies.

Supplementary Table S2. Anthropometric measurements pre, during and post pregnancy.

Time of data collection (N of pregnancies with anthropometric data)	Weight (kg)		Mean difference compared to pre-pregnancy (kg)	Height (cm)		BMI (kg/m²)	
	Mean ± SD	Median [range]		Mean ± SD	Median [range]	Mean ± SD	Median [range]
Pre-pregnancy (n=61)	70 ± 17	67 [45-140]	-	164 ± 6	164 [151-177]	26 ± 6	25 [18-53]
During pregnancy (n=91)	72 ± 16	70 [33-120]	+2 (median: +3)	163 ± 12	164 [151-180]	28 ± 16	26 [13-53]
Post-pregnancy (n=57)	73 ± 18	69 [48-136]	+3 (median: +2)	164 ± 6	163 [151-178]	27 ± 6	26 [19-44]

Abbreviations: Phe, phenylalanine; N, number; SD, standard deviation. Data on anthropometrics was collected yearly so no specifc data on trimesters or weight at baseline and end of pregnancy was available.
